# The King and the Salvation Army

**Published:** 1905-12-30

**Authors:** 


### THE KING AND THE SALVATION ARMY.

"GENERAL" BOOTH has received the following letter from the King, in response to his communication to his Majesty with respect to the Salvation Army's home colonising scheme :—

"December 21, 1905.

"Dear General Booth,—I am commanded by the King to thank you for your letter of the 19th inst., and to express to you at the same time his Majesty's great satisfaction at hearing of Mr. George Herring's magnificent donation, which will be so beneficial both to the home colonisation scheme of the Salvation Army and to the King's London Hospital Fund.

"His Majesty directs me to assure you that he shall watch with the greatest interest your important experiment, and it is hardly necessary to add that the undertaking has the King's most sincere sympathy and good wishes.—Believe me to be yours very truly, "KNOLLYS."

